# The Treatment of Tonsillitis

**Published:** 1893-01-14

**Authors:** 


### HULL ROYAL INFIRMARY.

### THE TREATMENT OF TONSILLITIS.

Acute tonsillitis in an adult, if seen in the early stage, is treated, in the first instance, by an emetic of ipecacuanha wine. When there is much swelling, pain in swallowing, and general febrile disturbance, the local treatment consists in gargling frequently with warm milk, and the inhalation of steam. A mustard poultice is applied to the outside of the neck for twenty minutes, then linseed poultices every four hours. Should the pain in swallowing and swelling of the tonsil increase, the head is held steady by an assistant, the tongue is depressed by a spatula, and a tenotomy knife is introduced into the prominent tonsil, and made to cut upwards and inwards, so as to relieve tension, and favour the evacuation of pus. A mixture of sodæ salicylatis gr.x., magnes. sulph. gr.xx., aq. ad ℥i. is ordered to be taken three times a day. The diet consists of food which can be swallowed without much effort, such as bread and milk, cornflour, arrowroot, gruel, broth, and soups, thickened with bread crumbs.

When the acute stage of inflammation is subsiding an astringent gargle is ordered, such as alum. gr. x., aquæ ℥i., ter die; and a tonic mixture, such as tinct. ferri perchlor., mx.; quiniæ sulph., gr.ij.; magnes. sulph., gr.xx.; aquam ad ℥i., t.d.s. Full diet is given, with wine or stout.

With regard to the treatment of chronic tonsillitis, excision is recommended in adults when there is much hypertrophy, and when frequent attacks of inflammation are complained of. It is also strongly urged in children when the tonsils continue prominent after repeated applications of astringents, where the voice is affected, and sleep disturbed from the obstruction to respiration.

An anæsthetic is given in the case of children, especially when there is deafness from the existence of adenoid vegetations, which are removed at the same time as the tonsils. There have been several troublesome cases of hæmorrhage after removal of the tonsils —children swallowing large quantities of blood and vomiting it afterwards, and adults requiring the successive application of hot-water pressure with a pad on the end of a sponge holder, the application of perchloride of iron, benzoline cautery, and subcutaneous injection of ergotine. The readiness with which ergot stops free oozing of blood has led to the administration of from a half dram to a dram of the liquid extract in water about ten minutes before the operation.

A guillotine of the French pattern is used owing to the ease with which the tonsil is secured and lifted out of its bed by the sliding fork. With regard to the position of the patient, if an anæsthetic is used the patient lies upon a table without a pillow, with the head hanging well over the edge. In this position any blood that oozes from the cut surfaces collects in the top of the pharynx and back of the nose instead of running down the throat.

A gag is introduced on the right side of the mouth, the tongue depressed with a spatula, and the left tonsil being well pressed in from the outside of the neck by an assistant, the guillotine is applied to the tonsil and the sliding fork pressed well home before the cutting blade is drawn.

In holding the guillotine only the ends of the first and second fingers are placed in the rings on the handle. If the fingers are introduced beyond the first joint the power of leverage is lost, and the guillotine cannot easily be pressed firmly against the tonsil.

The gag is then transferred to the left side of the mouth, the throat sponged, and the right tonsil removed. If on inspection it is found that only the surface of the tonsil has been shaved off, the greater portion of the remaining part is enucleated with the forefinger nail. The finger also explores the back of the nares, and any adenoid vegetations are scraped off with the nail or with Dalby's artificial nail, the ring-knife or post-nasal forceps being very seldom required.

If no anæsthetic is used, the patient is seated, the head steadied by an assistant, the tongue depressed, and the guillotine applied. The patient is then told to swallow, and whilst the tonsils are projecting during this movement the fork is pushed in and the blade drawn.

In exploring the pharynx afterwards without a gag between the jaws the finger is preserved from a possible squeeze by the teeth by wearing the large joint of Langenbeck's steel finger-guard.

It is necessary to remember to push the fork of the guillotine the full distance it can go into the tonsil, till the fork stands out from the ring-knife, as should the tonsil be tough, the patient make any resistance, and the operator draw the cutting blade prematurely, the points of the fork may be drawn into the circle of the sliding blade, and if sufficient force is used the blade may be broken.

If, on inspection, a small artery should be seen spouting from the cut surface of the tonsil, it is seized by a pair of clip forceps. Those with handles bent at a slight angle to the blades are most convenient. These are left on a minute or two, or a few twists may be given. The application of hot water, tannic acid, perchloride of iron, or pressure, are very seldom necessary when a dose of ergot has been given before the operation.

